# Sea-shell-like B_31_^+^ and B_32_: two new axially chiral members of the borospherene family†

**DOI:** 10.1039/d0ra01087a

**Published:** 2020-03-10

**Authors:** Ling Pei, Miao Yan, Xiao-Yun Zhao, Yue-Wen Mu, Hai-Gang Lu, Yan-Bo Wu, Si-Dian Li

**Affiliations:** Institute of Molecular Science, Shanxi University Taiyuan 030006 China ywmu@sxu.edu.cn luhg@sxu.edu.cn lisidian@sxu.edu.cn; Department of Chemical Engineering and Safety, Binzhou University Binzhou 256603 China

## Abstract

Since the discovery of the cage-like borospherenes *D*_2d_ B_40_^−/0^ and the first axially chiral borospherenes *C*_3_/*C*_2_ B_39_^−^, a series of fullerene-like boron clusters in different charge states have been reported in theory. Based on extensive global minimum searches and first-principles theory calculations, we present herein two new axially chiral members *C*_2_ B_31_^+^ (I) and *C*_2_ B_32_ (VI) to the borospherene family. B_31_^+^ (I) features two equivalent heptagons on the top and one octagon at the bottom on the cage surface, while B_32_ (VI) possesses two equivalent heptagons on top and two equivalent heptagons at the bottom. Detailed bonding analyses show that both sea-shell-like B_31_^+^ (I) and B_32_ (VI) follow the universal σ + π double delocalization bonding pattern of the borospherene family, with ten delocalized π bonds over a σ skeleton, rendering spherical aromaticity to the systems. Extensive molecular dynamics simulations show that these novel borospherenes are kinetically stable below 1000 K. The IR, Raman, and UV-vis spectra of B_31_^+^ (I) and B_32_ (VI) are computationally simulated to facilitate their future experimental characterizations.

## Abstract 2

Two new axially chiral sea-shell-like boron clusters *C*_2_ B_31_^+^ (a) and *C*_2_ B_32_ (b) are presented at first-principles theory level to the borospherene family.

## Graphical abstract

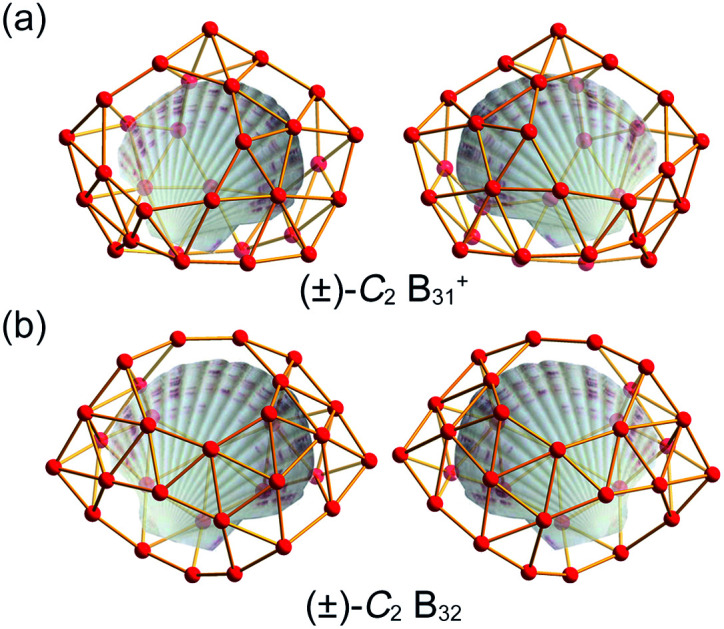

## 1. Introduction

As the lighter neighbour of carbon in the periodic table, boron is a typical electron-deficient element which shares with carbon the rare ability to form stable covalently bonded molecular frameworks with multicentre–two-electron bonds (mc–2e bonds) in both polyhedral molecules and bulk allotropes.^1,2^ Persistent joint photoelectron spectroscopy (PES) experimental and first-principles theory investigations by Lai-Sheng Wang and co-workers in the past two decades on size-selected negatively-charged boron clusters B_*n*_^−^ (*n* = 3–42) have revealed a rich landscape for boron nanoclusters from planar or quasi-planar (2D) structures (*n* = 3–38, 41, and 42) to cage-like borospherenes (*n* = 39, 40).^3–8^ The first all-boron fullerenes *D*_2d_ B_40_^−/0^, dubbed borospherenes, were discovered in 2014, marking the onset of borospherene chemistry.^5^ The spherically aromatic borospherene *D*_2d_ B_40_ is found to be composed of twelve interwoven boron double chains with two hexagons at the top and bottom and four heptagons on the waist. It features a unique bonding pattern of σ + π double delocalization, with twelve delocalized π bonds spherically distributed over a σ skeleton. The axially chiral B_39_^−^ appears to be the only boron cluster monoanion observed in experiments to date which has a cage-like global minimum (GM).^4^ The spherically aromatic borospherene family has been expanded by our group at first-principles theory level to the cage-like B_*n*_^*q*^ series (*n* = 36–42, *q* = *n* − 40) in different charge states which are all composed of twelve interwoven double chains with a σ + π double delocalization bonding pattern.^4,5,9–12^ Two lowest-lying cage-like *C*_s_ B_39_^+^ isomers in the same bonding pattern were also predicted in theory.^13^ Sea-shell-like *C*_2_ B_28_^−/0^ and *C*_s_ B_29_^−^ with nine delocalized π bonds over a σ-skeleton were later observed as minor isomers in PES experiments.^14,15^ Following the same structural motif, our group predicted the possibility of sea-shell-like *C*_s_ B_29_^+^, B_34_, and B_35_^+^ at first-principles theory levels^16,17^ which also appear to follow the σ + π double delocalization bonding pattern of the borospherene family. Ion mobility measurements in combination with density-functional theory (DFT) calculations, on the other hand, indicate that B_*n*_^+^ monocations possess double-ring tubular structures in the size-range between *n* = 16–25, showing another important structural domain for boron.^18^ However, the geometrical and electronic structures of B_*n*_^+^ monocations in the size range between *n* = 30–38 has remained unknown to date, except the sea-shell-like B_35_^+^ previously predicted by our group.^17^

In this work, we perform a theoretical investigation on the structures and bonding patterns of B_31_^+^ and B_32_*via* extensive global minimum searches and first-principles theory calculations. Sea-shell-like *C*_2_ B_31_^+^ (I) and *C*_2_ B_32_ (VI) are found to be the well-defined GMs of B_31_^+^ and B_32_, respectively, presenting two new axially chiral members to the borospherene family. Both B_31_^+^ (I) and *C*_2_ B_32_ (VI) appear to follow the universal σ + π double delocalization bonding pattern of the borospherene family, with ten delocalized π bonds over a σ skeleton, rendering spherical aromaticity to these novel borospherenes.

## 2. Theoretical procedure

**Fig. 1:**
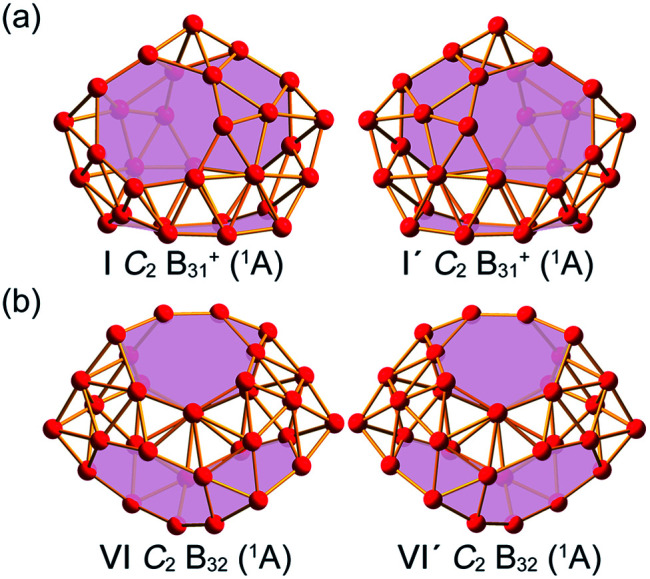
Optimized global minimum structures of the axially chiral borospherenes (a) *C*_2_ B_31_^+^ (I) and (b) *C*_2_ B_32_ (VI) and their degenerate enantiomers *C*_2_ B_31_^+^ (I′) and *C*_2_ B_32_ (VI′), with the B_7_ heptagons and B_8_ octagons on the cage surfaces highlighted in pink.

Extensive GM searches were performed on B_31_^+^ and B_32_ using the TGmin program,^19–21^ in conjunction with manual structural constructions based on the previously reported low-lying isomers of B_31_^−/0^ and B_32_^−/0^.^22^ About 5500 and 4500 trial points were generated on the potential energy surface for B_31_^+^ and B_32_ at the PBE/TZP level of theory. Frozen core approximation was used for the inner shells of [1s^2^] for B. The low-lying isomers for B_31_^+^ and B_32_ were then fully optimized at PBE0 and TPSSh levels^23,24^ with the 6-311+G(d) basis set,^25^ with vibrational frequencies checked to make sure all the low-lying isomers obtained were true minima. All these calculations were implemented using the Gaussian 16 program.^26^ To obtain more accurate relative energies, the top five lowest-lying isomers of B_31_^+^ and B_32_ were further refined at the single-point CCSD(T)/6-311G(d) level^27,28^ at their PBE0/6-311+G(d) geometries with the zero-point energy (ZPE) corrections included at PBE0. The obtained GMs *C*_2_ B_31_^+^ (I) and *C*_2_ B_32_ (VI) and their degenerated enantiomers *C*_2_ B_31_^+^ (I′) and *C*_2_ B_32_ (VI′) are shown in Fig. 1 and more low-lying isomers are listed in Fig. S1 (ESI).† Chemical bonding analyses on B_31_^+^ (I) and B_32_ (VI) (Fig. 1) were conducted using the Adaptive Natural Density Partitioning (AdNDP) method^29,30^ at the PBE0/6-31G level. Nucleus-independent chemical shifts (NICS)^31,32^ were calculated at the cage centres to assess the spherical aromaticity of B_31_^+^ (I) and B_32_ (VI). The IR and Raman spectra of B_31_^+^ (I) and B_32_ (VI) were simulated at PBE0/6-311+G(d) level and UV-vis absorption spectra calculated using the time-dependent DFT approach (TD-PBE0)^33,34^ implemented in Gaussian 16. Extensive Born–Oppenheimer molecular dynamics (BOMD) simulations were performed for B_31_^+^ (I) and B_32_ (VI) at 500 K, 700 K, and 1000 K for 30 ps (Fig. S2, ESI†) using the CP2K software,^35^ with the GTH-PBE pseudopotential and the DZVP-MOLOPT-SR-GTH basis set adopted.

## 3. Results and discussion

### 3.1. Structures and stabilities

**Fig. 2:**
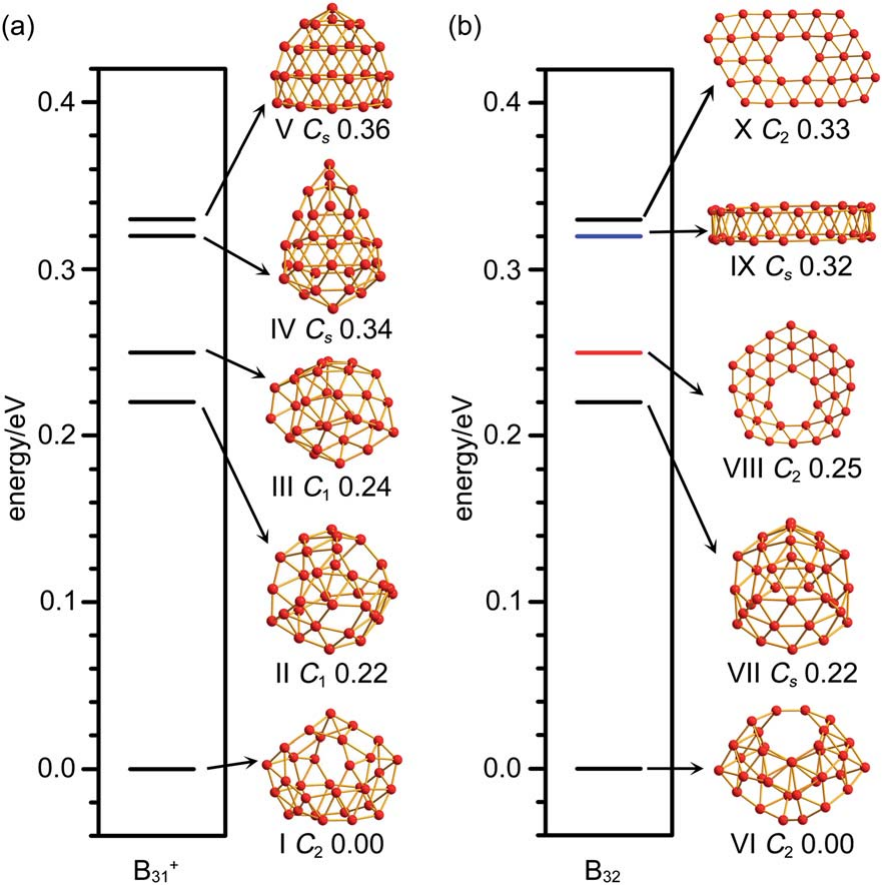
Configurational energy spectra of (a) B_31_^+^ and (b) B_32_ at CCSD(T)/6-311G(d)//PBE0/6-311+G(d) level, with the relative energies indicated in eV. Black, red and blue horizontal lines represent cage-like, quasi-planar and tubular structures, respectively.

As shown in the configurational energy spectrum of B_31_^+^ at the CCSD(T)/6-311G(d) level in Fig. 2a, the axially chiral sea-shell-like *C*_2_ B_31_^+^ (I) is the well-defined GM of B_31_^+^ with the lowest vibrational frequency of 66.2 cm^−1^. It consists of twenty-six triangles and eight quadrilaterals on the cage surface, two equivalent B_7_ heptagons on the waist, and one B_8_ octagon at the bottom (shaded in pink in Fig. 1a), following the Euler's rule in this case which reads: *E* (66 edges) = *F* (26 triangular + 8 quadrilaterals + 2 heptagonal + 1 octagonal faces) + *V* (31 vertices) − 2. The second, third, fourth, and fifth lowest-lying cage-like *C*_1_ B_31_^+^ (II), *C*_1_ B_31_^+^ (III), *C*_s_ B_31_^+^ (IV), and *C*_s_ B_31_^+^(V) lie 0.22, 0.24, 0.34, and 0.36 eV higher in energy than *C*_2_ GM at CCSD(T) level, respectively (Fig. 2a), though B_31_^+^ (III) lying 0.04 eV lower than the *C*_2_ GM at the less accurate PBE0 level (Fig. S1a, ESI†). The sea-shell-like low-symmetry *C*_1_ B_31_^+^ (II) possesses three heptagons on the surface, while *C*_1_ B_31_^+^ (III) contains two hexagons and one heptagon. We notice that most of the low-lying B_31_^+^ isomers are cage-like, whereas the first close-packed quasi-planar isomer *C*_1_ B_31_^+^ lies much higher (by 0.38 eV) than the GM at PBE0 (Fig. S1a, ESI†). The quasi-planar *C*_1_ B_31_^+^ with a hexagonal hole at the centre which corresponds to the GM of quasi-planar B_31_^−^ appears to be 0.63 eV higher than *C*_2_ GM at PBE0, well illustrating the charge-induced structural transition from planar B_31_^−^ reported in ref. 22 to cage-like B_31_^+^ obtained in this work due to two valence electrons' difference (Fig. S1a, ESI†).

*C*_2_ B_32_ (VI), the well-defined GM of neutral B_32_ at CCSD(T) level, also possesses an axially chiral sea-shell-like structure with the lowest vibrational frequency of 141 cm^−1^. It contains two equivalent heptagons on the top and two equivalent heptagons at the bottom on the cage surface (shaded in pink in Fig. 1b) and follows the Euler's rule which in this case reads: *E* (72 edges) = *F* (36 triangular + 2 quadrilaterals + 4 heptagonal faces) +*V* (32 vertices) − 2. The second lowest-lying *C*_s_ B_32_ (VII) possesses two hexagons on the waist and one octagon at the bottom (Fig. S1b, ESI†). It is worth noticing that the quasi-planar *C*_s_ B_32_ (VIII) predicted by Nguyen *et al.*^36^ and the double-ring tubular *D*_16d_ B_32_ (IX) proposed by Zhao's group^37^ in Fig. 2b turned out to be the third and fourth lowest-lying isomers of neutral B_32_ lying 0.25 eV and 0.32 eV higher in energy than our *C*_2_ GM (VI) at CCSD(T), respectively (Fig. 2b). B_32_ (VI) thus has the lowest energy in all the structures obtained to date for neutral B_32_. The much-concerned quasi-planar *C*_2_ B_32_ (X) with a B_6_ hexagon at the center which corresponds to the experimentally observed third isomer of *C*_2_ B_32_^−^ (ref. 22) appears to be 0.33 eV less stable than B_32_ (VI) at CCSD(T) (Fig. 2b).

Extensive molecular dynamics (MD) simulations were performed on B_31_^+^ (I) and B_32_ (VI) to check the dynamical stabilities of these axially chiral borospherenes. As shown in Fig. S2 (ESI),† both B_31_^+^ (I) and B_32_ (VI) appear to be dynamically stable between 500–1000 K. The calculated average root-mean-square-deviations (RMSD) and maximum bond length deviations (MAXD) of B_31_^+^ (I) are RMSD = 0.07, 0.11, and 0.13 Å and MAXD = 0.28, 0.46 and 0.58 Å at 500 K, 700 K, and 1000 K, respectively. The corresponding values of B_32_ (VI) turn out to be RMSD = 0.07, 0.08 and 0.10 Å and MAXD = 0.21, 0.27 and 0.36 Å at 500 K, 700 K, and 1000 K, respectively. No high energy isomers are observed in these MD simulation processes.

### 3.2. Bonding pattern analyses

**Fig. 3:**
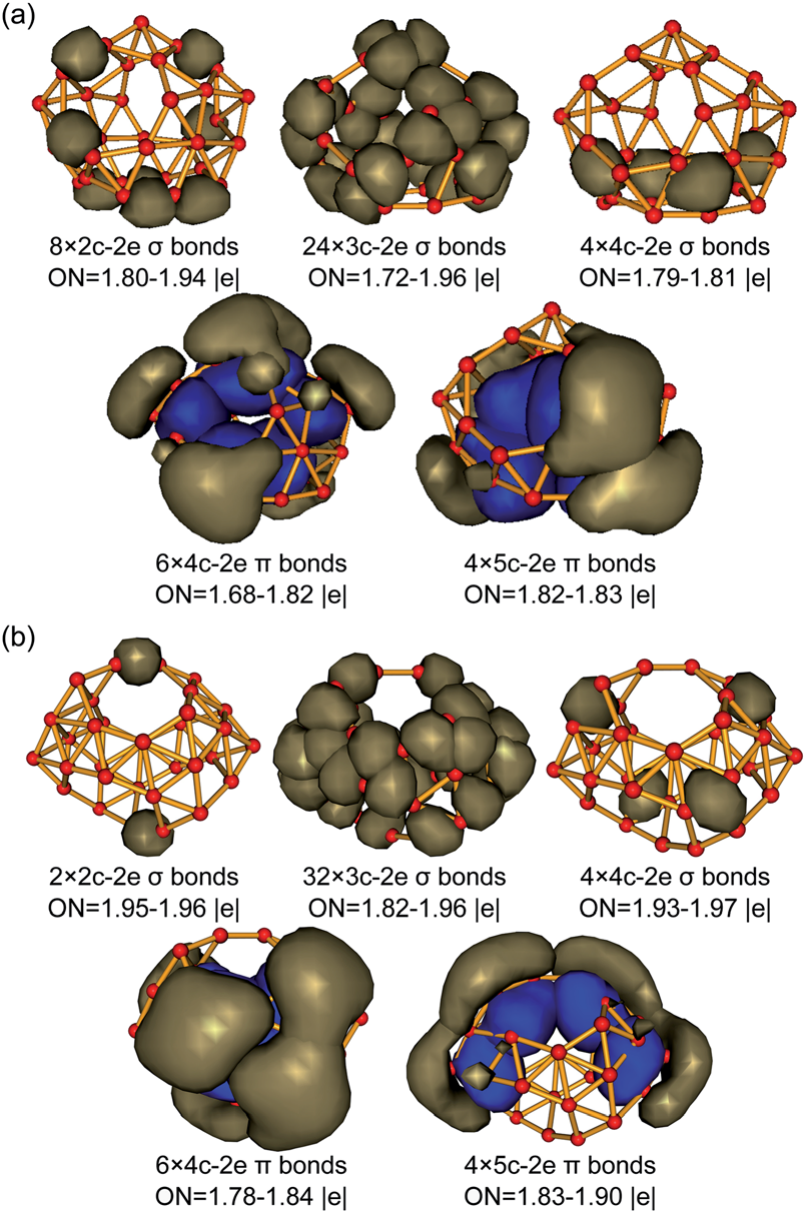
σ and π AdNDP bonding patterns of (a) *C*_2_ B_31_^+^ (I) and (b) *C*_2_ B_32_ (VI), with the occupation numbers (ONs) indicated.

The high thermodynamic and dynamic stabilities of these axially chiral borospherenes originate from their unique electronic structures and bonding patterns. We choose to use the widely used AdNDP approach developed by Boldyrev and co-workers to analyse both the localized and delocalized bonding interactions in these novel species.^29,30^ Detailed AdNDP analyses indicate that B_31_^+^ (I) possesses 8×2c–2e σ bonds, 24×3c–2e σ bonds and 4×4c–2e σ bonds on the cage surface with the occupation numbers of ON = 1.80–1.94 |*e*|, 1.72–1.96 |*e*|, and 1.79–1.81 |*e*|, respectively. The remaining 20 valence electrons form 10 delocalized π bonds spherically distributed over the σ-skeleton, including 6×4c–2e π bonds and 4×5c–2e π bonds with ON = 1.68–1.83 |*e*|, in an overall bonding symmetry of *C*_2_ (Fig. 3a). B_32_ (VI) possesses a similar bonding pattern with B_31_^+^ (I) (Fig. 3b). It contains 2×2c–2e σ bonds, 32×3c–2e σ bonds, and 4×4c–2e σ bonds on the cage surface. There exist 10 delocalized π bonds spherically distributed over the σ-skeleton, including 6×4c–2e π bonds and 4×5c–2e π bonds with ON = 1.78–1.90 |*e*|, in an overall symmetry of *C*_2_. Both B_31_^+^ (I) and B_32_ (VI) thus possess 10 delocalized π bonds over a σ-skeleton and follow the universal σ + π double delocalization bonding pattern of the borospherene family.^4,5,9–16^ Detailed bonding analyses further indicate that *C*_s_ B_32_ (VII), the second lowest-lying isomer of B_32_, also matches the σ + π double delocalization bonding pattern, with 10 delocalized π bonds over a σ skeleton (Fig. S3, ESI†).

The 10 delocalized π bonds on the cage surfaces renders spherical aromaticity to sea-shell-like B_31_^+^ (I) and B_32_ (VI), as evidenced by their calculated negative NICS values of NICS = −29 ppm and −24 ppm at the cage centers. We tabulate the numbers of σ bonds, π bonds, and calculated NICS values of the borospherene family reported so far in Table 1. It can be seen that the numbers of σ bonds increase monotonously with the number of valence electrons of the systems, while the numbers of π bonds increase in a stepwise pattern, with *C*_2_ B_28_, *C*_s_ B_29_^+^, and *C*_s_ B_29_^−^ possessing 9 delocalized π bonds, *C*_2_ B_31_^+^ and *C*_2_ B_32_ having 10 delocalized π bonds, *C*_2_ B_34_ and *C*_2_ B_35_^+^ containing 11 delocalized π bonds, and *T*_h_ B_36_^4−^, *C*_s_ B_37_^3−^, *C*_s_ B_38_^2−^, *C*_s_ B_39_^+^, *C*_3_ B_39_^−^, *C*_2_ B_39_^−^, *D*_2d_ B_40_, *C*_1_ B_41_^+^, and *C*_2_ B_42_^2+^ in different charge states possessing 12 delocalized π bonds, respectively. These borospherenes all appear to be spherically aromatic with the negative calculated NICS values of NICS = −21 to −43 ppm. It is these delocalized π bonds that help to maintain the cage-like structures of the borospherene family and render spherical aromaticity to the systems.

**Table 1:** The numbers of σ bonds (*n*_σ_), π bonds (*n*_π_), and calculated NICS (ppm) values of the borospherene family reported to date

	*n*_σ_	*n*_π_	NICS/ppm
*C*_2_ B_28_ (ref. 14)	33	9	−40
*C*_s_ B_29_^+^ (ref. 16)	34	9	−34
*C*_s_ B_29_^−^ (ref. 15)	35	9	−21
*C*_2_ B_31_^+^	36	10	−29
*C*_2_ B_32_	38	10	−24
*C*_2_ B_34_ (ref. 17)	40	11	−40
*C*_2_ B_35_^+^ (ref. 17)	41	11	−38
*T*_h_ B_36_^4−^ (ref. 12)	44	12	−36
*C*_s_ B_37_^3−^ (ref. 10)	45	12	−33
*C*_s_ B_38_^2−^ (ref. 11)	46	12	−37
*C*_s_ B_39_^+^ (ref. 13)	46	12	−40
*C*_3_ B_39_^−^ (ref. 4)	47	12	−38
*C*_2_ B_39_^−^ (ref. 4)	47	12	−39
*D*_2d_ B_40_ (ref. 5)	48	12	−43
*C*_1_ B_41_^+^ (ref. 9)	49	12	−41
*C*_2_ B_42_^2+^ (ref. 9)	50	12	−40

### 3.3. Spectral simulations

**Fig. 4:**
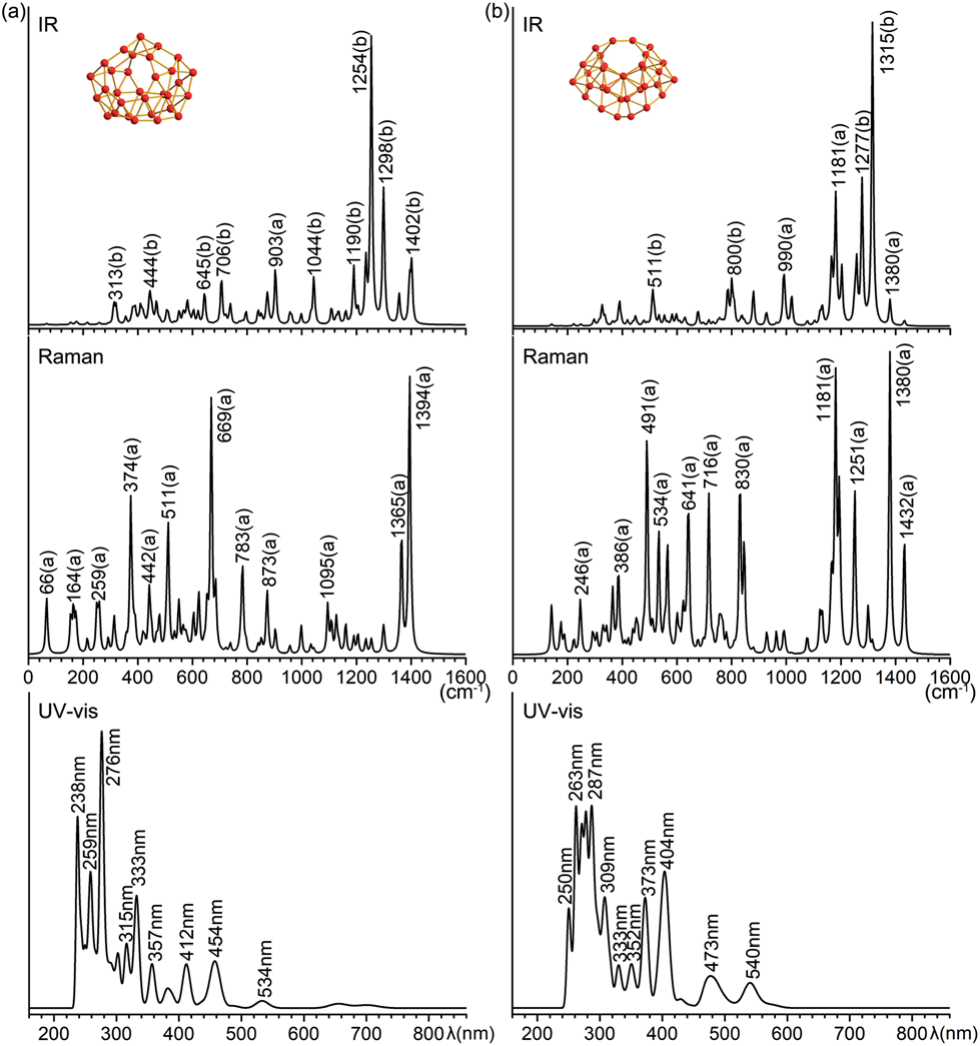
Simulated IR, Raman, and UV-vis absorption spectra of (a) *C*_2_ B_31_^+^ (I) and (b) *C*_2_ B_32_ (VI) at PBE0/6-311+G(d) level.

Infrared photodissociation (IR-PD) spectra in combination with first-principles theory calculations have proven to be an effective approach in characterizing novel clusters.^38^ B_31_^+^ (I) and B_32_ (VI) possess 87(43a + 44b) and 90(46a + 44b) vibrational modes, respectively. The simulated IR, Raman and UV-vis spectra of B_31_^+^ (I) and B_32_ (VI) are shown in Fig. 4. The major IR peaks of the two borospherenes appear to lie between 1100 and 1400 cm^−1^, with two major IR active peaks at 1254 cm^−1^ (b) and 1298 cm^−1^ (b) in B_31_^+^ (I) and two major peaks at 1277 cm^−1^ (b) and 1315 cm^−1^ (b) in B_32_ (VI). All the other IR vibrational modes appear to have much lower intensities. The major Raman active peaks occur at 374 cm^−1^ (a), 669 cm^−1^ (a) and 1394 cm^−1^ (a) in B_31_^+^ (I) and 491 cm^−1^ (a), 1181 cm^−1^ (a) and 1308 cm^−1^ (a) in B_32_ (VI), with main contributions originating from the symmetric vibrational modes. The Raman vibrational modes at 374 cm^−1^ (a) in B_31_^+^ (I) and 491 cm^−1^ (a) in B_32_ (VI) correspond to the typical “radial breathing modes” (RBMs) of the two borospherenes which can be used to characterize the hollow structures of single-walled boron nanoclusters in experiments.^39^

The simulated UV-vis spectra of B_31_^+^(I) and B_32_ (VI) lie between 200–550 nm, with the main absorption peaks lying at 238 nm, 276 nm, 333 nm, 412 nm, 454 nm, and 534 nm in B_31_^+^(I) and at 263 nm, 287 nm, 373 nm, 404 nm, 473 nm, and 540 nm in B_32_ (VI), respectively (Fig. 4). The strong UV-vis peaks originate from electronic excitations from the deep inner shells to the high-lying unoccupied molecular orbitals of the systems, while the weak absorption bands above 500 nm are attributed to electronic excitations from the occupied frontier orbitals (HOMO and HOMO−1) to the unoccupied frontier orbitals (LUMO, LUMO+1, and LUMO+2).

## 4. Summary

We have performed in this work an extensive first-principles theory investigation on sea-shell-like *C*_2_ B_31_^+^ (I) and *C*_2_ B_32_ (VI), presenting two new axially chiral members to the borospherene family. These novel borospherenes follow the universal σ + π double delocalization bonding pattern of the borospherene family, with 10 delocalized π bonds over an σ skeleton on the cage surface, rendering spherical aromaticity to these borospherene species. B_31_^+^ (I) may be characterized in gas-phases IR-PD spectral measurements, while B_32_ (VI) may be detected in matrix isolation infrared spectroscopy.^40^ More investigations on cage-like B_*n*_^+/0^ clusters are currently in progresses to further expand the borospherene family and enrich borospherene chemistry.

## Conflicts of interest

There are no conflicts to declare.

## Supplementary Material

RA-010-D0RA01087A-s001
