# Supplementary material for: Sea-shell-like B31+ and B32: two new axially chiral members of the borospherene family
Source: RSC Adv. 2020 Mar 10;10(17):10129–33. doi: 10.1039/d0ra01087a (PMC9050388; doi:10.1039/d0ra01087a)
Supplement: RA-010-D0RA01087A-s001 [file RA-010-D0RA01087A-s001.pdf]

Electronic Supplementary Material (ESI) for RSC Advances.
This journal is © The Royal Society of Chemistry 2020

Supporting Information

Sea-shell-like B_{31}^{+} and B_{32}: two new axially chiral members of the borospherene family

Ling Pei,[a,b] Miao Yan,[a] Xiao-Yun Zhao,[a] Yue-Wen Mu,[a] Hai-Gang Lu,[a] Yan-Bo Wu,[a] Si-Dian Li*[a]

Fig.S1 Low-lying isomers of B_{31}^{+} (a) and B_{32} (b), along with their relative energies indicated in eV at PBE0/6-311+G(d), TPSSh/6-311+G(d) (in parentheses), and CCSD(T)/6-311G(d)//PBE0/6-311+G(d) (in square brackets) levels. The energies are corrected for zero-point energies.

Fig.S2 Born-Oppenheimer molecular dynamics simulations of C_2 B_{31}^{+} (c) and C_2 B_{32} (d) at 500K, 700K and 1000K for 30 ps. The root-mean-square-deviation (RMSD) and maximum bond length deviation (MAXD) values (on average) are indicated in Å.

Fig. S3 σ and π AdNDP bonding patterns of C_s B_{32} (**VII**), with the occupation numbers (ONs) indicated.

Table S1 Optimized cartesian coordinates of the GM structures of B_{31}^{+} and B_{32} at PBE0/6-311+G(d)

Fig.S1 Low-lying isomers of B_{31}^+ (a) and B_{32} (b), along with their relative energies indicated in eV at PBE0/6-311+G(d), TPSSh/6-311+G(d) (in parentheses), and CCSD(T)/6-311G(d)//PBE0/6-311+G(d) levels. The energies are corrected for zero-point energies.

(a) B_{31}^+

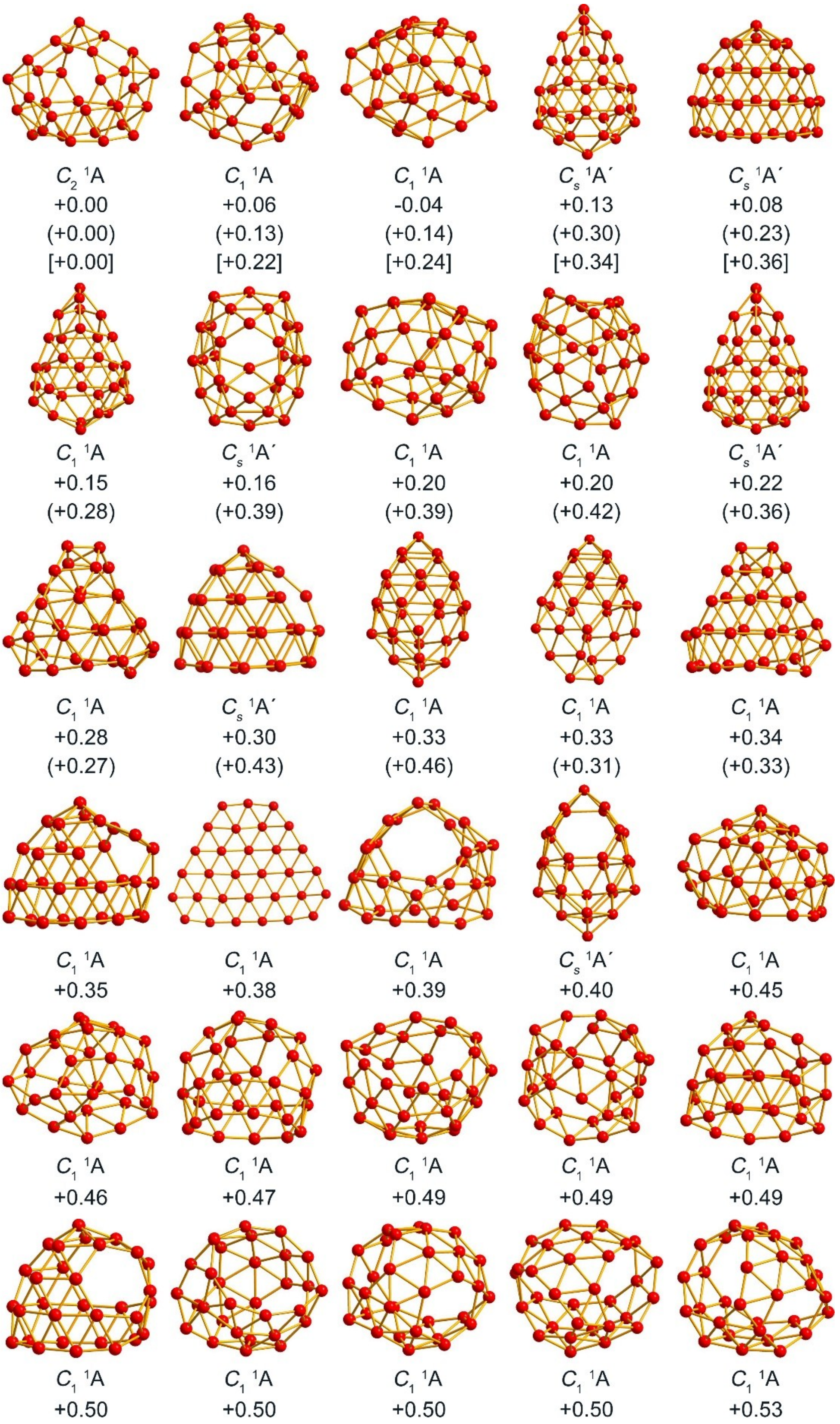

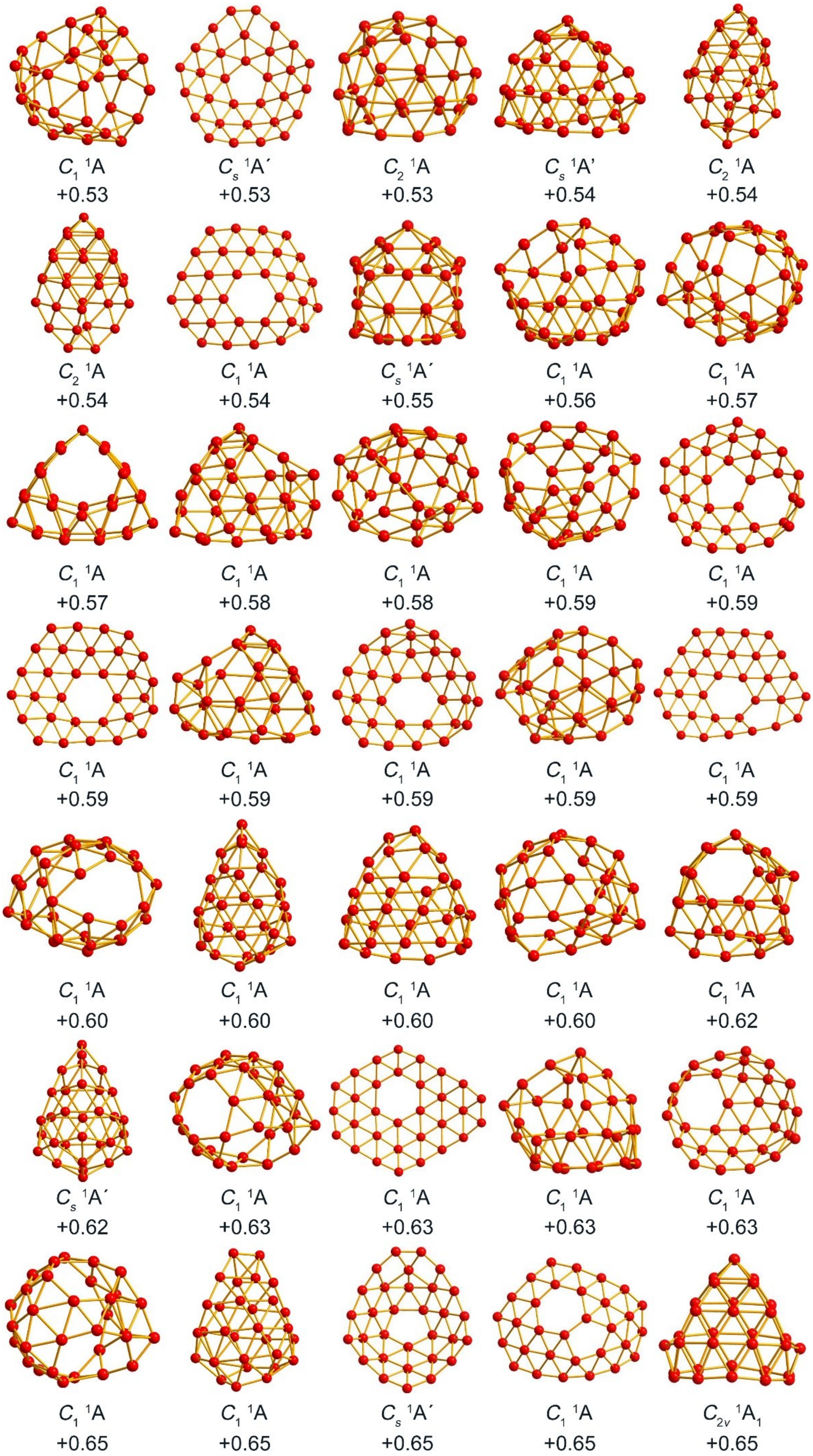
C_1 ^{1}A +0.53
C_s ^{1}A´ +0.53
C_2 ^{1}A +0.53
C_s ^{1}A' +0.54
C_2 ^{1}A +0.54
C_2 ^{1}A +0.54
C_1 ^{1}A +0.54
C_s ^{1}A´ +0.55
C_1 ^{1}A +0.56
C_1 ^{1}A +0.57
C_1 ^{1}A +0.57
C_1 ^{1}A +0.58
C_1 ^{1}A +0.58
C_1 ^{1}A +0.59
C_1 ^{1}A +0.59
C_1 ^{1}A +0.59
C_1 ^{1}A +0.59
C_1 ^{1}A +0.59
C_1 ^{1}A +0.59
C_1 ^{1}A +0.59
C_1 ^{1}A +0.60
C_1 ^{1}A +0.60
C_1 ^{1}A +0.60
C_1 ^{1}A +0.60
C_1 ^{1}A +0.62
C_s ^{1}A´ +0.62
C_1 ^{1}A +0.63
C_1 ^{1}A +0.63
C_1 ^{1}A +0.63
C_1 ^{1}A +0.63
C_1 ^{1}A +0.65
C_1 ^{1}A +0.65
C_s ^{1}A´ +0.65
C_1 ^{1}A +0.65
C_{2v} ^{1}A$_1$ +0.65

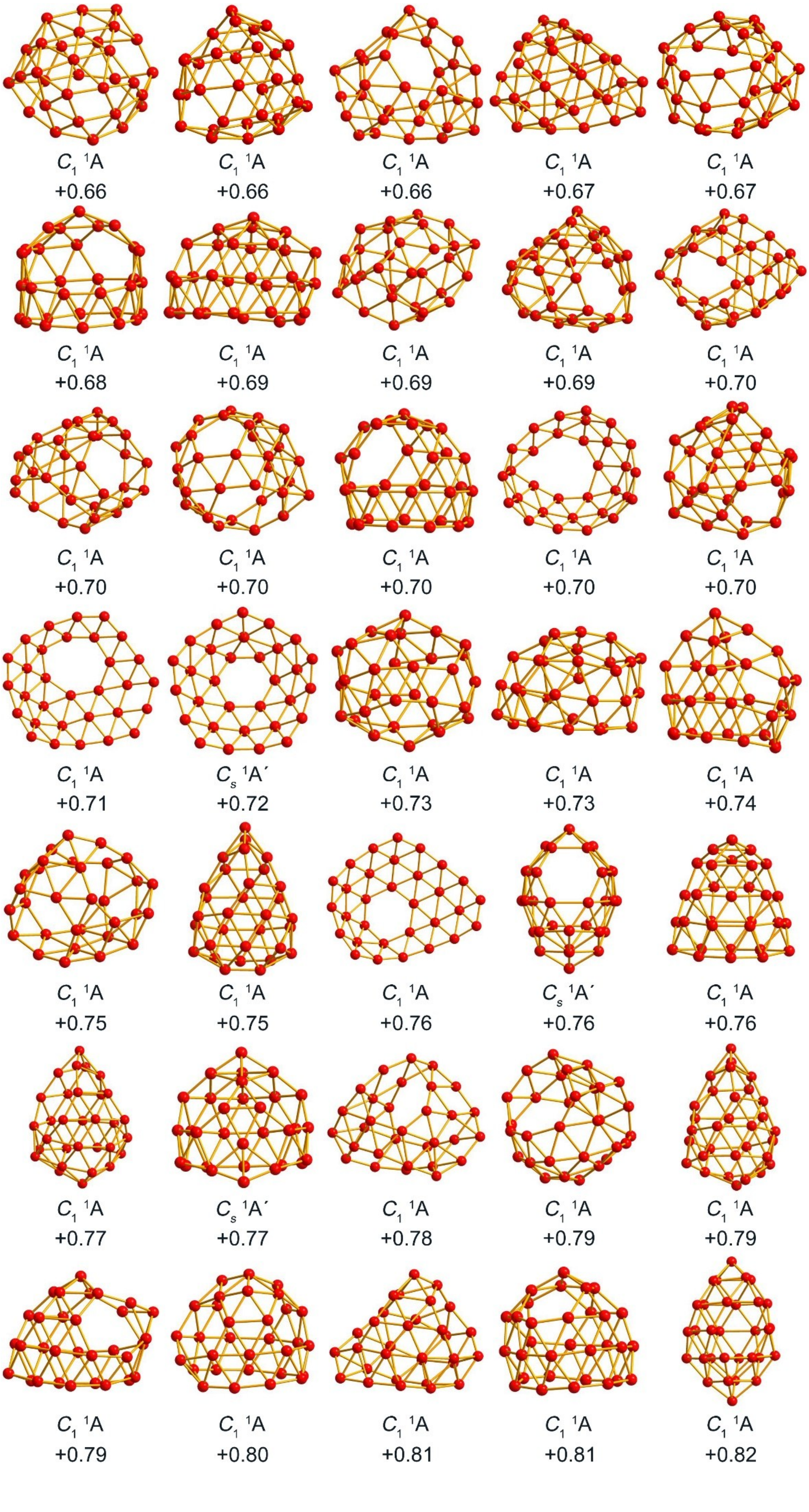

C_1 ^{1}A +0.66
C_1 ^{1}A +0.66
C_1 ^{1}A +0.66
C_1 ^{1}A +0.67
C_1 ^{1}A +0.67
C_1 ^{1}A +0.68
C_1 ^{1}A +0.69
C_1 ^{1}A +0.69
C_1 ^{1}A +0.69
C_1 ^{1}A +0.70
C_1 ^{1}A +0.70
C_1 ^{1}A +0.70
C_1 ^{1}A +0.70
C_1 ^{1}A +0.70
C_1 ^{1}A +0.70
C_1 ^{1}A +0.71
C_s ^{1}A′ +0.72
C_1 ^{1}A +0.73
C_1 ^{1}A +0.73
C_1 ^{1}A +0.74
C_1 ^{1}A +0.75
C_1 ^{1}A +0.75
C_1 ^{1}A +0.76
C_s ^{1}A′ +0.76
C_1 ^{1}A +0.76
C_1 ^{1}A +0.77
C_s ^{1}A′ +0.77
C_1 ^{1}A +0.78
C_1 ^{1}A +0.79
C_1 ^{1}A +0.79
C_1 ^{1}A +0.79
C_1 ^{1}A +0.80
C_1 ^{1}A +0.81
C_1 ^{1}A +0.81
C_1 ^{1}A +0.82

(b) B_{32}

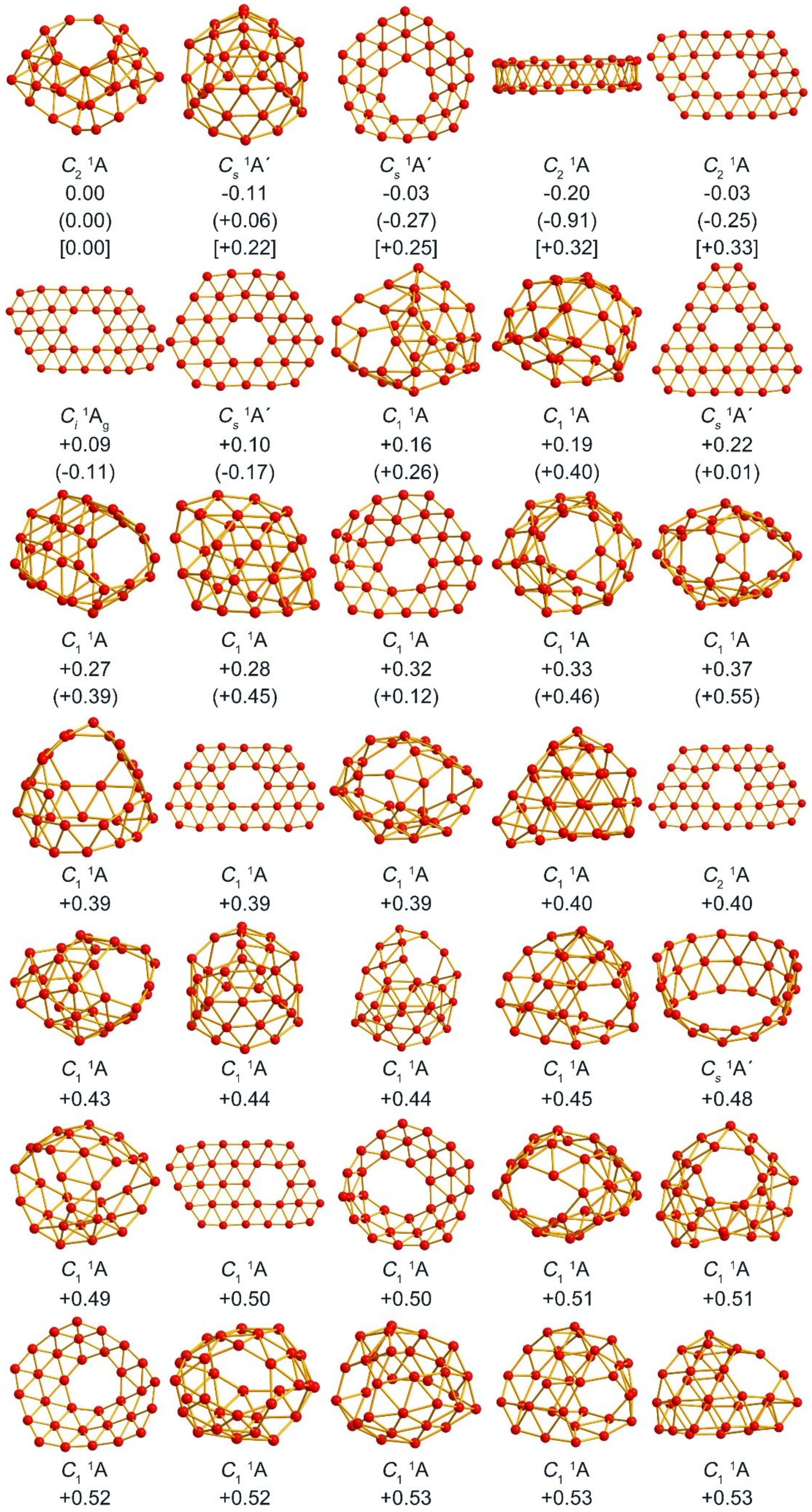
C2 1A
0.00
(0.00)
[0.00]
Cs 1A´
-0.11
(+0.06)
[+0.22]
Cs 1A´
-0.03
(-0.27)
[+0.25]
C2 1A
-0.20
(-0.91)
[+0.32]
C2 1A
-0.03
(-0.25)
[+0.33]
Ci 1Ag
+0.09
(-0.11)
Cs 1A´
+0.10
(-0.17)
C1 1A
+0.16
(+0.26)
C1 1A
+0.19
(+0.40)
Cs 1A´
+0.22
(+0.01)
C1 1A
+0.27
(+0.39)
C1 1A
+0.28
(+0.45)
C1 1A
+0.32
(+0.12)
C1 1A
+0.33
(+0.46)
C1 1A
+0.37
(+0.55)
C1 1A
+0.39
C1 1A
+0.39
C1 1A
+0.39
C1 1A
+0.40
C2 1A
+0.40
C1 1A
+0.43
C1 1A
+0.44
C1 1A
+0.44
C1 1A
+0.45
Cs 1A´
+0.48
C1 1A
+0.49
C1 1A
+0.50
C1 1A
+0.50
C1 1A
+0.51
C1 1A
+0.51
C1 1A
+0.52
C1 1A
+0.52
C1 1A
+0.53
C1 1A
+0.53
C1 1A
+0.53

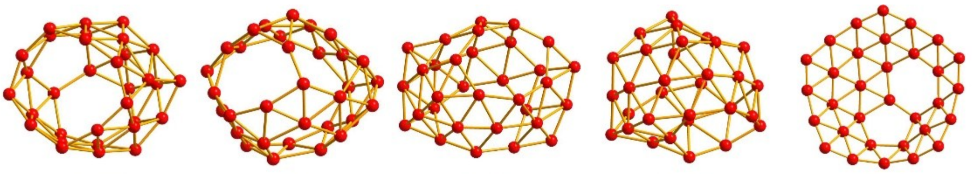

C_1 ^{1}A	C_1 ^{1}A	C_1 ^{1}A	C_1 ^{1}A	C_1 ^{1}A
+0.54	+0.54	+0.56	+0.57	+0.57

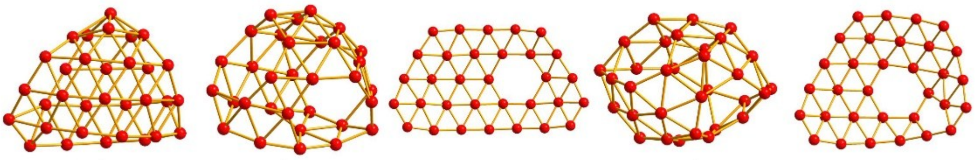

C_1 ^{1}A	C_1 ^{1}A	C_1 ^{1}A	C_1 ^{1}A	C_1 ^{1}A
+0.57	+0.58	+0.59	+0.59	+0.60

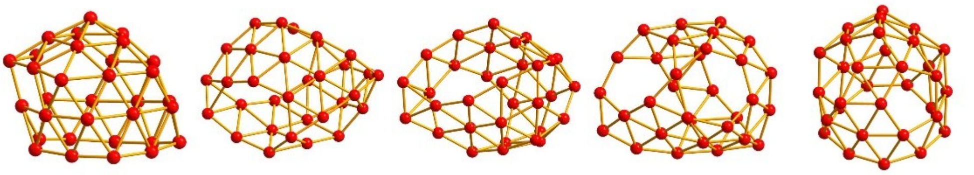

C_1 ^{1}A	C_1 ^{1}A	C_1 ^{1}A	C_1 ^{1}A	C_1 ^{1}A
+0.61	+0.63	+0.63	+0.64	+0.64

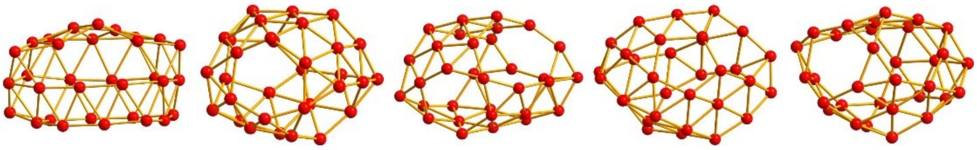

C_1 ^{1}A	C_1 ^{1}A	C_1 ^{1}A	C_1 ^{1}A	C_1 ^{1}A
+0.65	+0.65	+0.65	+0.67	+0.67

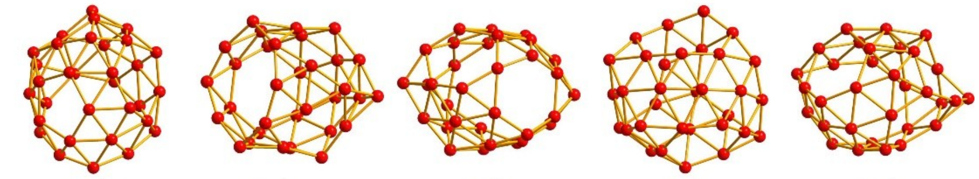

C_1 ^{1}A	C_1 ^{1}A	C_1 ^{1}A	C_1 ^{1}A	C_1 ^{1}A
+0.69	+0.69	+0.70	+0.70	+0.70

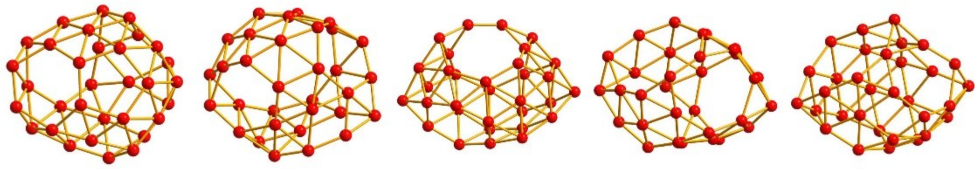

C_1 ^{1}A	C_1 ^{1}A	C_1 ^{1}A	C_1 ^{1}A	C_1 ^{1}A
+0.71	+0.71	+0.72	+0.72	+0.72

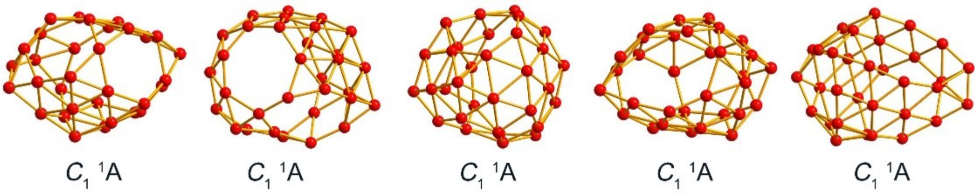

C_1 ^{1}A	C_1 ^{1}A	C_1 ^{1}A	C_1 ^{1}A	C_1 ^{1}A
+0.73	+0.73	+0.74	+0.76	+0.77

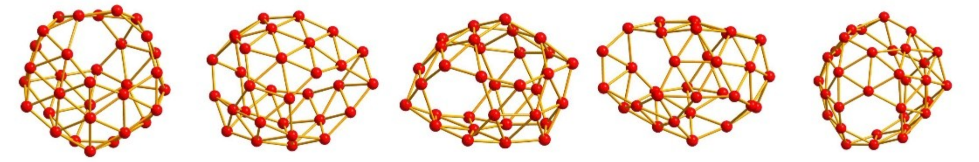

C_1 ^{1}A	C_1 ^{1}A	C_1 ^{1}A	C_1 ^{1}A	C_1 ^{1}A
+0.77	+0.78	+0.78	+0.80	+0.83

Fig.S2 Born-Oppenheimer molecular dynamics simulations of C_2 B_{31}^{+} (a) and C_2 B_{32} (b) at 500K, 700K and 1000K for 30 ps. The root-mean-square-deviation (RMSD) and maximum bond length deviation (MAXD) values (on average) are indicated in Å.

(a) C_2 B_{31}^{+}

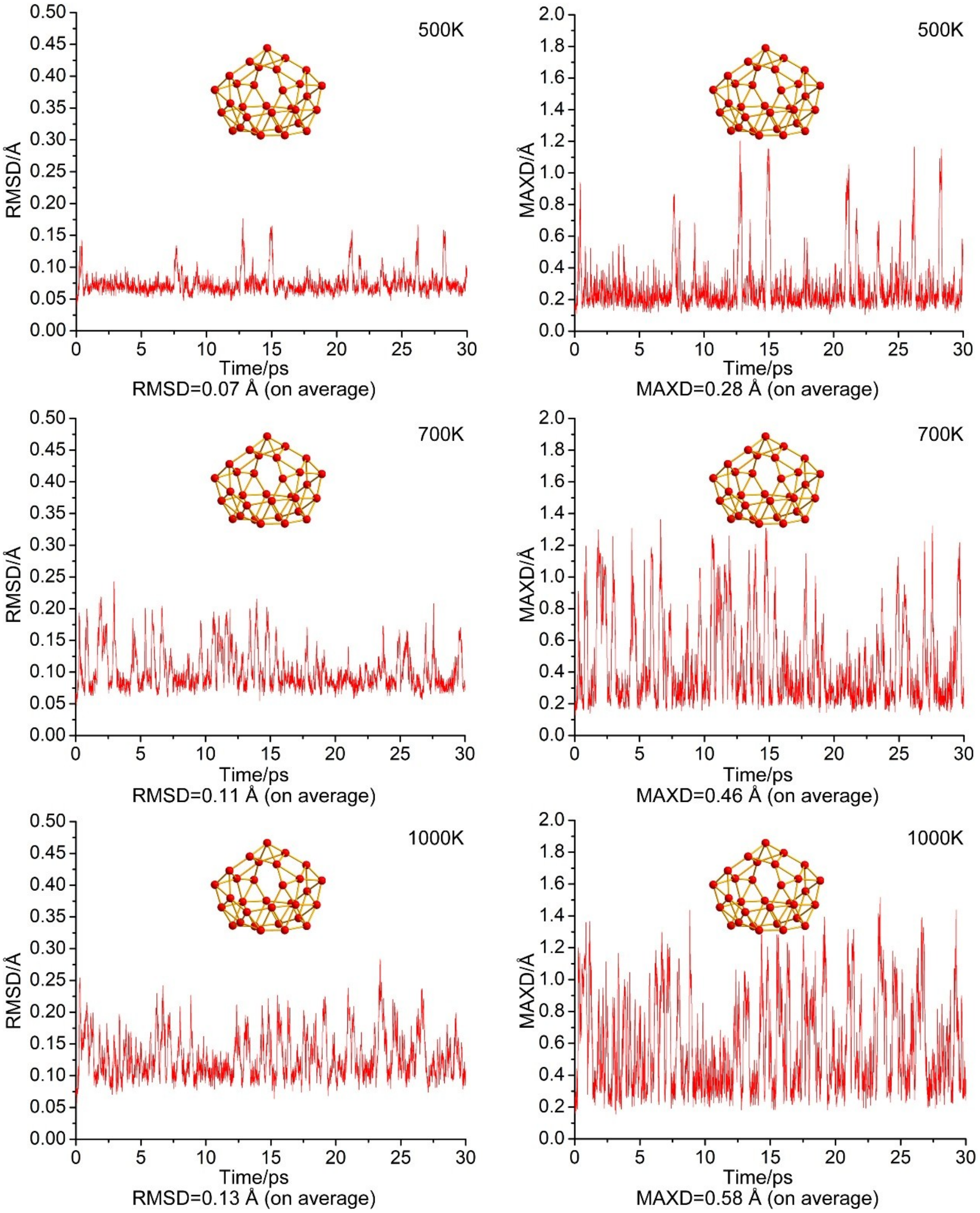

(b) C_2 B_{32}

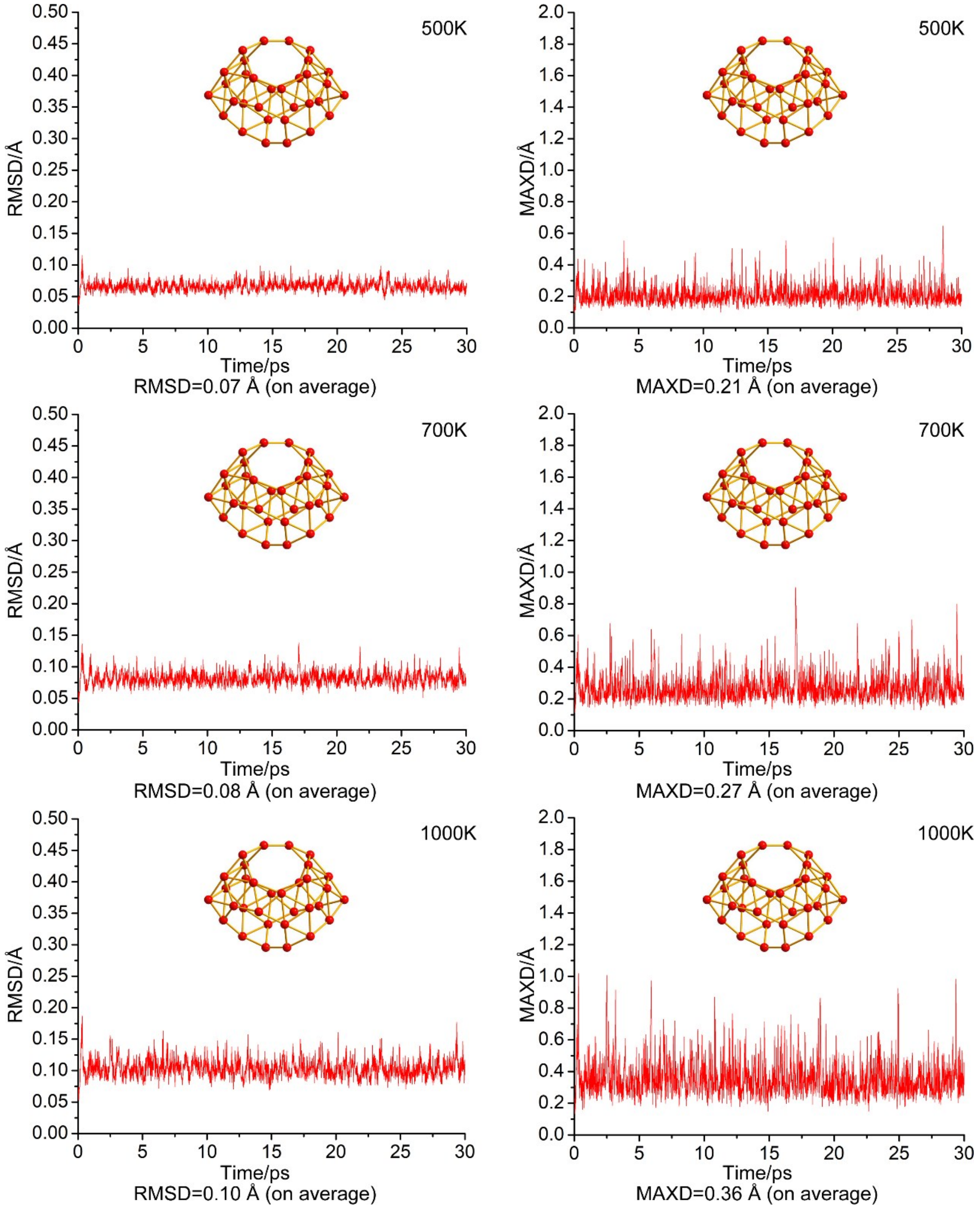
500K
RMSD/Å
Time/ps
RMSD=0.07 Å (on average)
500K
MAXD/Å
Time/ps
MAXD=0.21 Å (on average)
700K
RMSD/Å
Time/ps
RMSD=0.08 Å (on average)
700K
MAXD/Å
Time/ps
MAXD=0.27 Å (on average)
1000K
RMSD/Å
Time/ps
RMSD=0.10 Å (on average)
1000K
MAXD/Å
Time/ps
MAXD=0.36 Å (on average)

Fig. S3 σ and π AdNDP bonding patterns of C_s B_{32} (**VII**), with the occupation numbers (ONs) indicated.

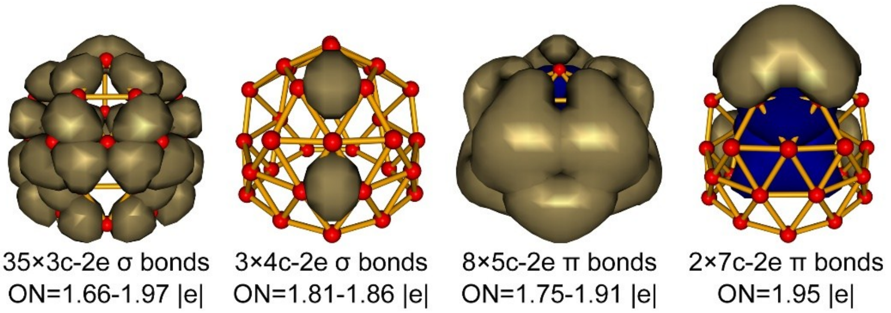

Table S1 Optimized cartesian coordinates of the GM structures of B_{31}^+ and B_{32} at PBE0/6-311+G(d)

1. C_2 B_{31}^+

B	-1.88265100	0.84149000	-0.61297700
B	-1.23583500	2.34274400	-0.84656300
B	1.04759800	0.08582100	2.05691600
B	-1.25600900	1.36700600	-2.10904800
B	1.85263300	1.72351000	-2.02279500
B	1.23583500	-2.34274400	-0.84656300
B	-0.23383800	-2.03425600	-1.66756000
B	1.25600900	-1.36700600	-2.10904800
B	1.72009700	0.16416000	-2.18178100
B	1.61518400	2.63926400	-0.73907300
B	0.35920600	2.65837700	1.75516700
B	0.00000000	2.85425400	0.00141400
B	1.58053200	1.47097100	1.04460400
B	1.99250200	0.85882700	-0.55731700
B	1.44478100	3.17331100	0.78737700
B	-2.04785500	0.06398100	0.79355600
B	1.88265100	-0.84149000	-0.61297700
B	0.00000000	-2.85425400	0.00141400
B	-1.85263300	-1.72351000	-2.02279500
B	0.23383800	2.03425600	-1.66756000
B	-1.72009700	-0.16416000	-2.18178100
B	-0.35920600	-2.65837700	1.75516700
B	-0.08757500	1.37087400	2.61018900
B	-1.61518400	-2.63926400	-0.73907300
B	-1.99250200	-0.85882700	-0.55731700
B	-1.58053200	-1.47097100	1.04460400
B	-1.44478100	-3.17331100	0.78737700
B	-1.04759800	-0.08582100	2.05691600
B	0.08757500	-1.37087400	2.61018900
B	2.04785500	-0.06398100	0.79355600
B	0.00000000	0.00000000	3.37578000

2. C_2 B_{32}

B	-1.21140200	1.89639800	2.32158200
B	-1.81198000	-1.70956800	0.97782200
B	1.24310500	-2.39542800	-0.54834700
B	-0.38349800	0.70519800	2.85201600
B	1.95187100	0.27981900	0.15532200
B	-1.60991000	-0.46586200	-1.58631900
B	-0.52354600	-0.59548300	-2.87848700
B	-0.91400200	2.93418600	1.09567500

B	-0.40123100	3.81366900	-0.19811100
B	0.79495600	2.83856900	0.43663700
B	0.40123100	-3.81366900	-0.19811100
B	1.81198000	1.70956800	0.97782200
B	1.63361200	-1.28840000	0.75860000
B	1.60991000	0.46586200	-1.58631900
B	1.21140200	-1.89639800	2.32158200
B	-0.40123100	-1.79546100	1.75140200
B	1.65607000	1.84967900	-0.66595100
B	1.15684900	1.90680400	-2.25473200
B	0.38349800	-0.70519800	2.85201600
B	0.40123100	1.79546100	1.75140200
B	-1.63361200	1.28840000	0.75860000
B	-1.15684900	-1.90680400	-2.25473200
B	-0.79495600	-2.83856900	0.43663700
B	0.27395400	2.98247900	-1.34049600
B	-1.65607000	-1.84967900	-0.66595100
B	-1.86330500	0.99269700	-0.87661400
B	-1.24310500	2.39542800	-0.54834700
B	-1.95187100	-0.27981900	0.15532200
B	1.86330500	-0.99269700	-0.87661400
B	0.52354600	0.59548300	-2.87848700
B	-0.27395400	-2.98247900	-1.34049600
B	0.91400200	-2.93418600	1.09567500
